# Effect of Anaerobic Calcium Oxide Alkalization on the Carbohydrate Molecular Structures, Chemical Profiles, and Ruminal Degradability of Rape Straw

**DOI:** 10.3390/ani13152421

**Published:** 2023-07-26

**Authors:** Jiayi Zhu, Fucan Li, Zeling Wang, Haitao Shi, Xi Wang, Yanling Huang, Shengli Li

**Affiliations:** 1 Key Laboratory of Qinghai-Tibetan Plateau Animal Genetic Resource Reservation and Utilization, Ministry of Education, Southwest Minzu University, Chengdu 610041, China; 2 State Key Laboratory of Animal Nutrition, Beijing Engineering Technology Research Center of Raw Milk Quality and Safety Control, China Agricultural University, Beijing 100193, China

**Keywords:** rape straw, CaO alkalization, carbohydrate nutritional values, in situ degradation, molecular structure

## Abstract

**Simple Summary:**

Rape straw has been used as livestock feedstuff in many countries, but the low digestibility caused by the high degree of lignification has limited its application in ruminant diets. Numerous techniques have been employed to enhance the nutritional quality of rape straw, including physical, chemical, and microbial methods. Among these, anaerobic alkaline storage is particularly effective in improving the straw’s nutritional value and preventing mold growth. To improve the utilization efficiency of rape straw, anaerobic calcium oxide alkalization was conducted, and advanced molecular spectroscopy was applied, to detect the internal molecular structural changes. The calcium oxide treatment increased the contents of soluble fiber, improved the ruminal degradability of neutral detergent fiber and acid detergent fiber, and decreased the contents of indigestible fiber. Alterations in cellulosic compounds’ spectral regions were highly correlated with the differences in carbohydrate chemical constituents and the ruminal digestibility of rape straw, which indicated that the infrared techniques could be used to evaluate the carbohydrate nutritive properties. The results of this study showed that calcium oxide treatment and anaerobic storage changed the molecular characteristics and improved the nutritive properties of rape straw.

**Abstract:**

To improve the utilization efficiency of rape straw, anaerobic calcium oxide (CaO) alkalization was conducted, and advanced molecular spectroscopy was applied, to detect the internal molecular structural changes. Rape straw was treated with different combinations of CaO (3%, 5%, and 7%) and moisture levels (50% and 60%) and stored under anaerobic conditions. We investigated the carbohydrate chemical constituents, the ruminal neutral detergent fiber (aNDF) and acid detergent fiber (ADF) degradation kinetics, and the carbohydrate molecular structural features. CaO-treated groups were higher (*p* < 0.05) for ash, Ca, non-fiber carbohydrate, soluble fiber, and the ruminal degradability of aNDF and ADF. In contrast, they were lower (*p* < 0.05) for the contents of aNDF, ADF, and indigestible fiber. With CaO levels rising from 3% to 7%, the content of aNDF and ADF linearly decreased (*p* < 0.05). CaO treatment and anaerobic storage changed the molecular characteristics, including structural parameters related to total carbohydrates (TC), cellulosic compounds (CEC), and structural carbohydrates (STC). Alterations in cellulosic compounds’ spectral regions were highly correlated with the differences in carbohydrate chemical constituents and the ruminal digestibility of rape straw. In summary, CaO treatment and anaerobic storage altered the molecular structural parameters of carbohydrates, leading to an enhancement in the effective degradability (ED) of aNDF and ADF in rape straw. From the perspective of processing cost and effectiveness, 5% CaO + 60% moisture could be suggested as a recommended treatment combination.

## 1. Introduction

Oilseed rape (*Brassica napus* ssp. *oleifera* L.) is widely cultivated in the world. Rape straw, as the main coproduct after harvesting, has been considered an abundant biomass resource [1]. However, the majority of the crop residues have been wasted and burned in the open field, which contributes to serious environmental and social problems like air pollution, resource waste, and fire disasters [2]. Rape straw has been used as livestock feedstuff in many countries, but the low digestibility caused by a high degree of lignification has limited its application in ruminant diets [3,4]. To improve its digestibility, many physical and chemical treatments including NaOH pretreatment, dilute acid pretreatment, microwave pretreatment, and other treatments have been applied on rape stalks [5,6]. However, most of the methods have not been widely accepted by farmers due to their high processing costs, unsatisfying effectiveness, and environmental concerns [7]. According to a published study, CaO compared to other chemical reagents like NaOH presents lesser health risks and is easier to handle [8]. Recent studies have demonstrated the potential of anaerobic CaO alkalization in improving the nutritive value of crop residues and other roughages [9,10].

Fourier transform infrared spectroscopy (FTIR) is suggested as a fast and non-destructive method for investigating feed physiochemical characteristics and has been used in many fields such as food science and plant science [11,12]. FTIR can be divided into transmission, transflection, and attenuated total reflection (ATR) according to its spectroscopic sampling mode [13]. Recently, this technique has been applied to detect the molecular structure of the nutrients in different feedstuffs [14,15]. The molecular structural features of transgenic alfalfa and barley have been successfully determined by FTIR in recent studies [16,17,18].

It is still uncertain whether anaerobic CaO alkalization treatment could change the chemical composition and molecular features of carbohydrates in rape straw. Therefore, this research aimed to investigate (1) the carbohydrate chemical constituents, (2) the carbohydrate subfractions, (3) the ruminal aNDF and ADF degradation kinetics, (4) the carbohydrate molecular structural features, and (5) correlations between the nutritive properties and molecular characteristics of rape straw treated with different levels of CaO and moisture content.

## 2. Materials and Methods

### 2.1. Rape Straw Treatment

Rape straw (variety: Chuanyou 44) samples were obtained from the model agricultural garden located in the Xindu District of Chengdu city. The rape was manually divided into straw and rape pod shells, cut into 2–3 cm after the harvest, and then dried at 65 °C for further treatments. Treatments included three CaO levels (3%, 5%, and 7% of dry stover) and two moisture contents (50% and 60%). Calcium oxide (Sinopharm Chemical Reagent Co., Ltd., Shanghai, China, ≥98.0%) was suspended in water, and the slurry was mixed sufficiently with the straw in a drum. The straw was then compacted into sealed 2.5 kg capacity plastic bags to achieve a packing density of approximately 240 kg of DM/m^3^ and stored at room temperature (around 25 °C), evacuated, and heat sealed using a vacuum packaging machine (Lvye DZ280; Dongguan Yijian Packaging Machinery Co., Ltd., Dongguan, China). The vacuumed straw was kept for 3 weeks at ambient temperature [7]. Afterward, the bags were unsealed, and the straw was dried at 65 °C and ground for the analysis of the chemical profile (1 mm screen), rumen degradability (2 mm screen), and molecular structure (0.1 mm screen).

### 2.2. Chemical Analysis

In this study, the contents of dry matter (DM), crude protein (CP), ether extract (EE), and crude ash (Ash) were determined according to methods 934.01, 990.03, 920.39, and 924.05 of AOAC [19], respectively. The content of organic matter (OM) was determined by subtracting the ash content from the total percentage (100%). The Ankom fiber analyzer (Ankom Technology Corp., Macedon, NY, USA) was used to measure the contents of neutral detergent fiber (aNDF), acid detergent fiber (ADF), and acid detergent lignin (ADL) according to the Van Soest’s method [20], and α-amylase and sodium sulfite were used for the aNDF procedure. Contents of non-fiber carbohydrate (NFC) and total carbohydrate (CHO) were evaluated based on the NRC [21], where NFC = CHO − aNDF, hemicellulose = aNDF − ADF, cellulose = ADF − ADL, and CHO = 100 − CP − ash − EE.

### 2.3. Carbohydrate Fraction Partitioning

The carbohydrate subfractions were partitioned according to the Cornell Net Carbohydrate and Protein System (CNCPS 6.5) into the fractions of the rapid degradation of carbohydrates (CA), starch (CB1), soluble fiber (CB2), digestible fiber (CB3), and indigestible fiber (CC) [22,23]. Fraction CA is further divided into CA1 (volatile fatty acids), CA2 (lactic acid), CA3 (other organic acids), and CA4 (water-soluble carbohydrate). Fraction CC is indigestible fiber, which is the unavailable cell wall, and is assumed to be unavailable to animals [24]. The CNCPS carbohydrates fractions were calculated as follows:

CA(%CHO) = [100 − Starch(%NSC)] × [100 − CB2(%CHO) − CC(%CHO)]/100

CB2(%CHO) = 100 × {[NDF(%DM) − NDICP(%DM) − ADL(%DM) × 2.4]/CHO(%DM)}

CBl(%CHO) = Starch(%NSC) × [100 − CB2(%CHO) − CC(%CHO)]/100

CC(%CHO) = 100 ×[ADL(%DM) × 2.4/CHO(%DM)

### 2.4. In Situ Ruminal Degradation

All experimental procedures were approved by the Animal Management Committee (in charge of animal welfare issues) of Southwest Minzu University (SMU, Chengdu, China; approval code: SMU-AVS-22060101; approval date: 6 June 2022) and performed according to the ARRIVE guidelines for reporting animal research. Three healthy ruminal cannulated Holstein cows were employed for estimating rumen degradability by using an in situ method. All animals were managed according to the Code of Practice for the Housing and Care of Animals Used in Scientific Procedures [25]. The detailed procedure was conducted based on previously published literature [7,24]. The cows were fed thrice daily (07:30, 14:30, and 18:30), and water was available ad libitum. The diet was balanced according to the NRC maintenance requirement of dairy cattle [21]. Five grams of each sample in coded nylon bags (8 cm × 12 cm) were incubated in the rumens and removed after 0, 4, 8, 12, 24, 48, and 72 h. After the removal, the bags were rinsed in cold water to remove extra ruminal contents until the rinse water was clear and subsequently dried at 60 °C for 48 h. The pulverized samples were used for analyzing the ruminal degradation parameters of aNDF and ADF, which were determined based on Ørskov and McDonald [26] and modified by Robinson et al. [27]:

$$y=a+b(1-e^{-ct})$$

ED=a+[(b ∗ c)/(c+k)]

where *y* stands for the rumen disappearance of the incubated sample at *t* h; *a* is a rapidly degradable fraction, while *b* stands for slowly degradable components (%); *c* stands for the degradation rate of slowly degradable fraction (% h^−1^); *d* is the potentially degradable fraction in rumen degradation, *d* = *a* + *b*; *t* is the incubation time (h); *ED* is the in situ effective degradability of the incubated samples; *k* is the passage rate of the digest from the rumen (% h^−1^), which was assumed to be 0.031 in this study [28].

### 2.5. Molecular Spectroscopy on Carbohydrate Structure

An FTIR-7600 spectrometer (Lambda Scientific, Adelaide, Australia) was used for analyzing the carbohydrate molecular features. The mid-infrared spectra (ca. 4000–800 cm^−1^) were generated with 64 coadded scans, and each sample (0.1 mm rape straw) was collected four times with a spectral resolution of 4 cm^−1^. OMNIC software (8.2) was applied for identifying the carbohydrate molecular spectral data by analyzing the relationship between spectral bands and CHO-structure-related functional groups based on previous publications [29,30,31]. The partitions of carbohydrate-related molecular spectral bands are as follows: (1) peak area of TC region (TCA, ca. 1189–909 cm^−1^), which is mainly related to total CHO, and three peaks can be found at 1147 (TC1), 1093 (TC2), and 1029 (TC3) cm^−1^; (2) cellulosic compounds area (CECA, ca. 1292–1189 cm^−1^), which is mainly associated with cellulosic compounds, and one major peak can be observed at ca. 1240 cm^−1^; (3) structural carbohydrate area (STCA, ca. 1487–1189 cm^−1^), which is related to hemicellulosic and cellulosic compounds, and four major peaks can be found at ca. 1461 (STC1), 1423 (STC2), 1371 (STC3), and 1324 (STC4) cm^−1^.

### 2.6. Statistical Analysis

The MIXED procedure of SAS software (9.2) was used to analyze the data of the carbohydrate chemical profile, degradation kinetics, and molecular structure [32]. The Shapiro–Wilk test was performed to check the normal distribution of data. The model used for analysis was *Y_ijk_* = *μ* + α*_i_* + β*_j_* + (αβ)*_ij_* + ε*_ijk_*, where *Y_ijk_* is the observation of the dependent variable *ij*; *μ* is the overall population mean; α*_i_* is the fixed effect of CaO doses (3%, 5%, and 7%); β*_j_* is the fixed effect of the moisture levels (50% and 60%); (αβ)*_ij_* is the interaction between CaO doses and moisture; ε*_ijk_* is the random error associated with the observation *ijk*. A contrast statement was used to compare the difference between CaO and moisture levels. The Tukey–Kramer method was used to compare the means, and the significance level was set as *p* < 0.05. Orthogonal polynomial contrasts were conducted to explore the linear and quadratic effects of CaO levels (3%, 5%, and 7%). Rank correlations were performed since the data for the correlation study were not normally distributed. The PROC CORR of SAS with an option of SPEARMAN was applied for quantifying the molecular parameters about the chemical profiles and ruminal degradation kinetics.

## 3. Results

### 3.1. CHO Chemical Profile of CaO-Treated Rape Straw

The chemical constituents of CaO-treated rape straw are listed in Table 1. The physical characteristics of the treated straw were similar to the common silage despite the different colors. The CaO level and moisture were found to have significant effects on the CHO chemical profile (*p* < 0.05) except for P. There were interactions between moisture and CaO levels on the contents of most constituents, apart from NFC, aNDF, ADL, aNDF/OM, and ADL/OM. The contents of ash, EE, Ca, and NFC increased (*p* < 0.05) with CaO levels rising from 3% to 7%, whereas OM content, the aNDF/OM ratio, and the ADF/OM ratio linearly decreased (*p* < 0.05). Compared with the untreated group, lower ADL content was observed in the CaO-treated rape straw.

### 3.2. CHO Fraction Partitioning of CaO-Treated Rape Straw

The carbohydrate fractions, which were partitioned into CB2, CB3, and CC in this study by CNCPS, are presented in Table 2. The results showed that the contents of CB2, CB3, and CC were significantly affected by moisture and the CaO level. As the CaO level rose, CB2 content quadratically increased (*p* < 0.05), while the contents of CB3 and CC decreased linearly (*p* < 0.05). CB2, CB3, and CC represent soluble fiber, digestible fiber, and indigestible fiber (unavailable lignin-bound cell wall content), respectively.

### 3.3. Ruminal Degradability

Table 3 shows the effects of the CaO treatment on the rumen degradation characteristics of aNDF and ADF. The results showed that all in situ parameters were affected (*p* < 0.05) by the CaO level, and the interactions between moisture and the CaO level were found (*p* < 0.05). The ED of aNDF and ADF increased quadratically (*p* < 0.05), while the degradation rate of the slowly degradable fraction (fraction c) of aNDF and ADF decreased quadratically (*p* < 0.05) with the increasing CaO level. The highest ED of aNDF (36.76%) and ADF (36.58%) was observed in the rape stalks treated with 5% CaO and 60% moisture, and the untreated rape stalks had the lowest ED of aNDF and ADF among the treatments (24.39% and 24.79%, respectively).

### 3.4. Carbohydrate Molecular Structural Features

**Figure 1:**
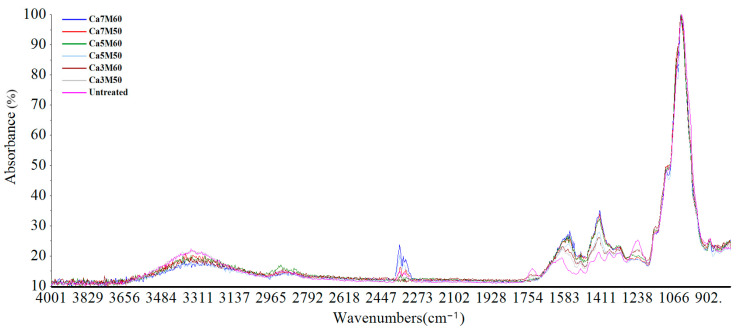
Fourier transform infrared spectroscopy spectra of the untreated and CaO-treated rape straw (Ca7M60: 7% CaO+60% moisture; Ca7M50: 7% CaO+50% moisture; Ca5M60: 5% CaO+60% moisture; Ca5M50: 5% CaO+50% moisture; Ca3M60: 3% CaO+60% moisture; Ca3M50: 3% CaO+50% moisture).

**Figure 2:**
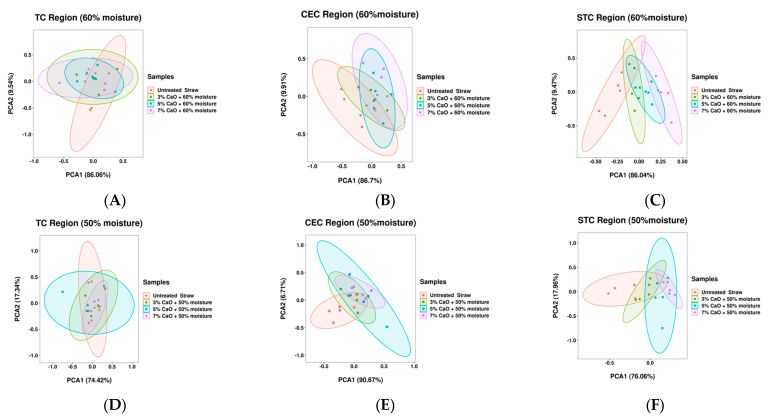
(**A**): Principal component analyses of the entire molecular spectral data from TC (total carbohydrate) region (1189–909 cm^−1^): comparison of the untreated and CaO-treated rape straw (3% CaO + 60% moisture; 5% CaO+60% moisture; 7% CaO+60% moisture). (**B**): Principal component analyses of the entire molecular spectral data from CEC (cellulosic compounds) region (1292–1189 cm^−1^). (**C**): Principal component analyses of the entire molecular spectral data from STC (structural carbohydrate) region (1487–1189 cm^−1^). (**D**): Principal component analyses of the entire molecular spectral data from TC region: comparison of the untreated and CaO-treated rape straw (3% CaO + 50% moisture; 5% CaO + 50% moisture; 7% CaO + 50% moisture). (**E**): Principal component analyses of the entire molecular spectral data from CEC region. (**F**): Principal component analyses of the entire molecular spectral data from STC region.

In order to further probe whether the alkali treatment had caused damage to the cellulose molecular structure of the rape straw, the FTIR analysis was carried out. The FTIR spectra of rape straw are shown in Figure 1, and the effects of CaO treatment on the CHO molecular structural features are presented in Table S1. The molecular structure parameters of TC2, TC1A, CEC, CECA, STC1, STC2, and STCA were affected by the CaO level (*p* < 0.05), and no interaction was observed between moisture and CaO level on all parameters of peak height and area values. As the CaO level rose, STC1 and STC2 were quadratically raised (*p* < 0.05), while CEC, CECA, and STCA decreased quadratically (*p* < 0.05). However, no difference was observed in STC1 and STC2 between the rape stalks treated with 5% CaO and 7% CaO. Figure 2A–F display the plots of the principal component analyses (PCA) of the three carbohydrate sub-regions (TC, CEC, and STC). The PCA plots of the TC region plotted the untreated and CaO-treated rape straw together. The CEC and STC regions showed clear differentiation between the untreated and CaO-treated groups with minimal overlaps, particularly in the rape stalks treated by 7% CaO and the untreated group. The first principal component of the TC, CEC, and STC regions interpreted 86.06%, 74.42%, 86.7%, 90.67%, 86.04%, and 76.06% of population variances.

### 3.5. Correlations between Carbohydrate Nutritive Properties and Molecular Structural Features

Table 4 and Table 5 present the relationships between the carbohydrate nutritive properties and molecular spectral parameters of rape straw. In our study, STC1 and STC2 were negatively related to the contents of aNDF and ADF (*p* < 0.01), while TCA, CEC, and CECA had the opposite tendency. In the CEC region (CEC and CECA), significant positive correlations were found with the content of ADF with r = 0.81 and 0.91 (*p* < 0.01), respectively. Strong negative correlations were found between the NFC content and TCA (r = −0.70, *p* = 0.01), CEC (*r* = −0.74, *p* < 0.01), and CECA (r = −0.86, *p* < 0.05). In general, the results showed that the CECA and TCA were correlated with most of the CHO parameters (*p* < 0.05) except for hemicellulose in rape stalks. Additionally, strong positive relationships were observed between CECA and the contents of CHO (r = 0.96, *p* < 0.01), ADF (r = 0.91, *p* < 0.01), aNDF (r = 0.96, *p* < 0.01), cellulose (r = 0.89, *p* < 0.01), and CB3 (r = 0.96, *p* < 0.01), while a strong negative correlation was found between CECA and CB2 (r = −0.91, *p* < 0.01).

The ruminal degradation kinetics of aNDF and ADF were also correlated with CHO molecular spectral parameters. In this study, the in situ ED of aNDF and ADF were positively related to TC1, TC2, STC1, and STC2 (*p* < 0.05) and negatively correlated with TCA, CEC, CECA, and STCA (*p* < 0.05). The degradation rates of the slowly degradable fractions (c fraction) of aNDF and ADF were positively correlated with CECA (*p* < 0.01, r = 0.73 and 0.71, respectively). STC1 and STC2 were found to have strong positive relationships with most in situ degradation parameters (*p* < 0.05). On the contrary, CEC and CECA were found to have correlations with most of the in situ aNDF and ADF degradation parameters (*p* < 0.05), and almost all these correlations were negative. Additionally, the data in Table 4 showed that strong positive correlations could be found between the parameters of the CEC region and ADF content.

## 4. Discussion

Our results were consistent with those of previous studies on corn stover in which a CaO treatment led to a decrease in OM content [7]. The decrease in OM content could be explained by the increases in ash content after CaO treatment. This fit in with the study of Zaman and Owen [33], which showed that the content of Ca and ash increased with the addition of Ca(OH)_2_. It is noteworthy that the increase in ash in treated rape straw did not correspond exactly to the added dose of CaO. Further studies are encouraged to explore the exact end-product of the alkalization and evaluate the OM loss during the processing. Research on the alkali treatment of fibrous materials has been conducted in many studies. Aslaniyan et al. [10] have conducted a study on soybean straw treated with NaOH, and they reported that NaOH alkalization changed the fiber composition, which was in accordance with our findings. He et al. [9] treated sea buckthorn (*Hippophae rhamnoides*) with CaO and observed decreased ADL content in CaO alkalization groups. They concluded that the reduced ADL content was mainly resulted from the alkali treatment, which could disrupt the plant cell wall by solubilizing hemicelluloses, lignin, and silica. Our results confirmed that CaO treatment could reduce the content of indigestible fiber and increase the proportion of NFC in rape straw. The decreased ADL content in CaO-treated rape straw might benefit the performance of animals. Based on changes in the chemical constituents and CNCPS contents of carbohydrates, the treatment combination of 5% CaO and 60% moisture might be an economical and effective condition for rape straw.

The results in Table 3 agreed with a previous study, which treated soybean straw with NaOH and CaO [10]. The authors found that the treated groups achieved a greater ruminal degradability of nutrients than the untreated group at almost all incubations. Shi et al. [7] reported that anaerobic CaO alkalization could improve the NDF ruminal degradability of corn stover. The main reason might be associated with the breaking of the chemical bonds of lignocellulosic when performing an alkali treatment, which increased the contact area with rumen microorganisms and improved the degradability of the corn stover. The results of the in situ study suggested that both CaO and moisture level had a significant influence on the ruminal degradability of aNDF and ADF. However, under the 60% moisture, both the ED values of aNDF and ADF were lower for the 7% CaO group compared to the 5% CaO group. This might be mainly attributed to the complex interaction between the CaO dose and the moisture levels, which needs to be further investigated. Considering the processing cost and effectiveness, the combination of 5% CaO and 60% moisture might be an economical treatment for rape straw. It is worth noting that the feeding value of the forage can also be affected by many other factors (e.g., the palatability, physical properties, etc.). Additionally, the elevated calcium content of the treated straw should not be ignored as an excessive amount of Ca in feed can pose challenges in feed formulations [7]. Further studies with animal performance data are highly needed to determine the optimal treatment conditions.

Because of the negligible amounts of sugar in stover, the spectral band assignments in the present study involved only the TC region (total carbohydrate), CEC region (cellulose compounds), and STC region (hemicellulosic and cellulosic compounds) [15]. Alterations in carbohydrate molecular structures are usually associated with changed chemical profiles and nutrient digestibility [34]. Previous studies reported that the alterations in carbohydrate structural parameters could be correlated with the nutritive properties [35,36]. A previous study conducted by Lei et al. [34] reported that CEC height was negatively correlated with ADF content in alfalfa, which was different from our findings. These discrepancies might have originated as a result of many factors such as the material types, treatment methods, etc. The results in Table 4 showed that the CECA and TCA were correlated with most of the CHO parameters. The previous literature on alfalfa and dry distiller grains with solubles (DDGS) reported similar observations [34,37]. Our results indicated that CECA was highly correlated with the carbohydrate chemical composition, which was consistent with another previous study [38].

Previous studies have shown that the molecular structure of carbohydrates can affect the rumen degradation rate of NDF in alfalfa and carinata meal [30,39]. The results in Table 5 were not in accordance with a published study that was conducted on Brassica carinata seed [40]. The discrepancy in the test material (carinata seed vs. rape straw) and the treatment methods might be responsible for the difference. The results indicated that the changes in the STC region might contribute to the variations in the degradation characteristics, which were consistent with the previous study [41]. Our study demonstrated that there were strong correlations between the ruminal NDF degradability and specific spectral data (e.g., the CEC and CECA). The results meant that the CEC region parameters might be used as an indicator to evaluate the in situ degradation characteristics of aNDF and ADF. Righi et al. (2017) explored the feasibility of using NIR to predict undigested NDF at 240 h of fermentation (uNDF240) in feeds. Li et al. (2015) reported that both the A_CELC/A_CHO ratio and the A_CELC/A_StCHO ratio were negatively correlated with uNDF240 [42]. Further studies could be conducted to explore whether it is possible to construct an FTIR model for predicting the ED or uNDF240 of the treated straw.

## 5. Conclusions

CaO treatment and anaerobic storage have significantly changed the carbohydrate nutritive properties and molecular structural makeup of the rape straw. The CaO treatment increased the contents of ash, Ca, NFC, and soluble fiber; improved the ruminal degradability of aNDF and ADF; and decreased the contents of OM, aNDF, ADF, and indigestible fiber. Moreover, according to the correlation study, the alterations in cellulosic compounds’ spectral regions were highly related to the differences in carbohydrate chemical constituents and the in situ degradability of the rape straw, which indicated that the CECA and STC could be used to evaluate the carbohydrate chemical composition and the degradation characteristics. In conclusion, the anaerobic alkalization could be used as an effective technique to change the chemical composition of carbohydrate and improve the ruminal digestibility of rape straw.

## Figures and Tables

**Table 1:** Chemical profile of rape straw treated with different CaO dosages and moisture levels ^1^.

Item	Untreated Straw	3% CaO		5% CaO		7% CaO		SEM ^2^	*p*-Value			Contrast ^3^		
		50% M ^4^	60% M	50% M	60% M	50% M	60% M		Moisture	CaO	M’CaO	Control vs. Treated	L	Q
Basic Chemical Profiles (% of DM)														
CP	2.72 ^a^	2.59 ^b^	2.47 ^c^	2.45 ^c^	2.50 ^c^	2.35 ^d^	2.48 ^c^	0.009	0.11	<0.01	<0.01	<0.01	<0.01	<0.01
Ash	5.53 ^e^	10.4 ^d^	10.5 ^d^	13.0 ^c^	13.8 ^b^	15.2 ^a^	15.1 ^a^	0.058	<0.01	<0.01	<0.01	<0.01	<0.01	<0.01
OM	94.5 ^a^	89.6 ^b^	89.5 ^b^	87.0 ^c^	86.2 ^d^	84.8 ^e^	84.9 ^e^	0.058	<0.01	<0.01	<0.01	<0.01	<0.01	<0.01
EE	0.46 ^bc^	0.41 ^c^	0.54 ^b^	0.39 ^c^	0.67 ^a^	0.70 ^a^	0.76 ^a^	0.016	<0.01	<0.01	<0.01	<0.01	<0.01	<0.01
Ca	1.04 ^e^	2.99 ^d^	2.84 ^d^	4.33 ^c^	4.35 ^c^	5.72 ^b^	6.02 ^a^	0.036	0.10	<0.01	<0.01	<0.01	<0.01	<0.01
P	0.09 ^abc^	0.10 ^ab^	0.09 ^bc^	0.09 ^abc^	0.09 ^abc^	0.08 ^c^	0.10 ^a^	0.003	0.17	1.00	<0.01	1.00	1.00	1.00
Carbohydrate Chemical Profiles (% of DM)														
NFC	7.08 ^c^	10.9 ^b^	9.94 ^b^	10.6 ^b^	10.2 ^b^	13.7 ^a^	15.3 ^a^	0.428	0.78	<0.01	0.07	<0.01	<0.01	0.32
ADF	68.8 ^a^	57.8 ^c^	60.2 ^b^	57.9 ^c^	55.7 ^cd^	54.5 ^d^	53.6 ^d^	0.561	0.03	<0.01	<0.01	<0.01	<0.01	<0.01
aNDF	84.3 ^a^	75.9 ^b^	76.5 ^b^	73.5 ^c^	72.8 ^c^	68.1 ^d^	66.4 ^d^	0.377	0.12	<0.01	0.07	<0.01	<0.01	0.01
ADL	13.2 ^a^	11.5 ^bcd^	12.0 ^b^	11.7 ^bc^	12.3 ^b^	10.8 ^d^	11.1 ^cd^	0.137	<0.01	<0.01	0.31	<0.01	<0.01	0.14
aNDF/OM	80.3 ^a^	76.8 ^b^	77.0 ^b^	76.6 ^b^	76.2 ^b^	72.3 ^c^	71.0 ^c^	0.325	0.09	<0.01	0.14	<0.01	<0.01	0.04
ADF/OM	65.2 ^a^	58.7 ^b^	62.7 ^a^	59.3 ^b^	58.3 ^b^	57.9 ^b^	55.0 ^c^	0.465	0.97	<0.01	<0.01	<0.01	<0.01	0.02
ADL/OM	12.8 ^a^	12.2 ^ab^	12.1 ^ab^	11.7 ^bc^	11.6 ^bc^	11.8 ^abc^	10.9 ^c^	0.190	0.05	0.01	0.08	<0.01	<0.01	0.22

^1^ Means with different letters within a row are significantly different at *p* < 0.05. ^2^ SEM, Standard error of the mean. DM, Dry matter; CP, crude protein; EE, ether extract; OM, organic matter; NFC, non-fiber carbohydrate; aNDF, neutral detergent fiber; ADF, acid detergent fiber; ADL, acid detergent lignin. Carbohydrate was calculated as 100 − EE − CP − Ash according to NRC-2001. ^3^ L = Linear; Q = quadratic. ^4^ M = Moisture.

**Table 2:** Carbohydrate fraction partitioning of rape straw treated with different CaO dosages and moisture levels ^1^.

Item	Untreated Straw	3%		5%		7%		SEM ^2^	*p*-Value			Contrast ^4^		
		50% M ^5^	60% M	50% M	60% M	50% M	60% M		Moisture	CaO	M’CaO	Control vs. Treated	L	Q
CNCPS Fractions (%CHO) ^3^														
CB2	8.03 ^e^	11.0 ^d^	11.5 ^cd^	12.6 ^c^	12.3 ^c^	16.7 ^b^	18.7 ^a^	0.235	0.01	<0.01	0.01	<0.01	<0.01	<0.01
CB3	57.4 ^a^	55.7 ^ab^	55.1 ^b^	54.1 ^bc^	52.3 ^cd^	51.6 ^d^	48.8 ^e^	0.344	<0.01	<0.01	0.04	<0.01	<0.01	0.11
CC	35.4 ^a^	34.6 ^ab^	33.4 ^abc^	33.3 ^bc^	31.8 ^c^	31.7 ^c^	32.5 ^c^	0.369	<0.01	<0.01	0.29	0.01	<0.01	0.67

^1^ Means with different letters within a row are significantly different at *p* < 0.05. ^2^ SEM, Standard error of the mean. ^3^ CNCPS, Cornell net carbohydrate and protein system. The subfractions of CHO partitioned by CNCPS included the following: CB2, soluble fiber (intermediately degradable carbohydrate fraction); CB3, digestible fiber (available neutral detergent fiber or slowly degradable carbohydrate fraction); CC, indigestible fiber (unavailable neutral detergent fiber). ^4^ L = Linear; Q = quadratic. ^5^ M = Moisture.

**Table 3:** Effect of different CaO dosages and moisture levels on ruminal degradation parameters of rape straw ^1^.

Item	Untreated Straw	3%		5%		7%		SEM ^2^	*p*-Value			Contrast ^3^		
		50%M ^4^	60%M	50% M	60% M	50% M	60% M		Moisture	CaO	M’CaO	Control vs. Treated	L	Q
In Situ Rumen DM Degradation														
*a*	3.78 ^e^	6.19 ^d^	9.43 ^c^	9.67 ^c^	12.9 ^a^	9.27 ^c^	11.4 ^b^	0.157	<0.01	<0.01	0.01	<0.01	<0.01	<0.01
*b*	17.4 ^c^	41.3 ^b^	51.0 ^ab^	59.1 ^a^	39.6 ^b^	42.6 ^b^	59.0 ^a^	2.197	0.25	0.17	<0.01	<0.01	<0.01	<0.01
*c*	0.06 ^a^	0.02 ^bcd^	0.01 ^d^	0.01 ^d^	0.03 ^b^	0.02 ^bc^	0.02 ^cd^	0.002	0.24	0.02	<0.01	<0.01	<0.01	<0.01
*d*	21.1 ^d^	47.5 ^c^	60.5 ^ab^	68.7 ^a^	52.5 ^bc^	51.8 ^bc^	70.3 ^a^	2.300	0.03	0.03	<0.01	<0.01	<0.01	<0.01
*ED*	15.3 ^c^	21.3 ^b^	22.1 ^b^	28.2 ^a^	30.9 ^a^	28.1 ^a^	30.9 ^a^	1.010	0.04	<0.01	0.54	<0.01	<0.01	0.01
In Situ Rumen aNDF Degradation														
*a*	6.81 ^e^	8.67 ^d^	10.7 ^c^	10.1 ^c^	14.8 ^a^	13.3 ^b^	10.1 ^c^	0.147	<0.01	<0.01	<0.01	<0.01	<0.01	<0.01
*b*	24.4 ^f^	36.9 ^e^	35.3 ^e^	47.4 ^c^	52.1 ^b^	43.0 ^d^	56.5 ^a^	0.348	<0.01	<0.01	<0.01	<0.01	<0.01	<0.01
*c*	0.08 ^a^	0.03 ^b^	0.03 ^bcd^	0.03 ^cd^	0.02 ^de^	0.03 ^b c^	0.02^e^	0.001	<0.01	<0.01	0.01	<0.01	<0.01	<0.01
*d*	31.3 ^d^	45.5 ^c^	46.0 ^c^	57.5 ^b^	66.9 ^a^	56.3 ^b^	66.6 ^a^	0.370	<0.01	<0.01	<0.01	<0.01	<0.01	<0.01
*ED*	24.4 ^e^	27.5 ^d^	27.4 ^d^	31.3 ^c^	36.8 ^a^	34.5 ^b^	31.1 ^c^	0.280	0.02	<0.01	<0.01	<0.01	<0.01	<0.01
In Situ Rumen ADF Degradation														
*a*	3.16 ^e^	9.23 ^d^	10.5 ^c^	12.9 ^b^	13.5 ^b^	15.6 ^a^	14.0 ^b^	0.220	0.62	<0.01	<0.01	<0.01	<0.01	<0.01
*b*	31.3 ^e^	38.1 ^c^	33.7 ^d^	50.5 ^a^	51.7 ^a^	43.1 ^b^	44.4 ^b^	0.378	0.08	<0.01	<0.01	<0.01	<0.01	<0.01
*c*	0.07 ^a^	0.03 ^b^	0.03 ^b^	0.02 ^c^	0.02 ^bc^	0.03 ^b^	0.02 ^bc^	0.002	0.34	<0.01	0.03	<0.01	<0.01	<0.01
*d*	34.4 ^e^	47.3 ^c^	44.2 ^d^	63.4 ^a^	65.2 ^a^	58.7 ^b^	58.4 ^b^	0.427	0.17	<0.01	<0.01	<0.01	<0.01	<0.01
*ED*	24.8 ^e^	28.6 ^cd^	27.7 ^d^	29.3 ^c^	36.6 ^a^	36.3 ^a^	33.3 ^b^	0.272	<0.01	<0.01	<0.01	<0.01	<0.01	0.01

^1^ Means with different letters within a row are significantly different at *p* < 0.05. *a*: The rapid degradable fraction in rumen degradation; *b*: the slowly degradable fraction in rumen degradation; *c*: the degradation rate of the slowly degradable fraction; *d*: the potentially degradable fraction in rumen degradation, *d* = *a* + *b*; *ED*: the effective degradability of the incubated samples. ^2^ SEM, Standard error of the mean. ^3^ L = Linear; Q = quadratic. ^4^ M = Moisture.

**Table 4:** Correlation between the carbohydrate molecular profiles and related nutrients in rape straw ^1^.

Item		Carbohydrate Chemical Profile (% of DM)							CNCPS Fractions (% of CHO)		
		CHO	NFC	ADF	aNDF	Hemicellulose	Cellulose	ADL	CB2	CB3	CC
TC1	r	−0.30	0.30	−0.21	−0.37	0.24	−0.28	0.01	0.28	−0.28	0.09
TC2	r	−0.56 *	0.50	−0.49	−0.62 *	0.11	−0.51	−0.23	0.57*	−0.59 *	−0.05
TC3	r	−0.10	−0.06	−0.06	−0.08	−0.12	−0.08	0.28	0.03	−0.19	0.44
TC1A	r	−0.27	0.35	−0.21	−0.37	0.18	−0.25	−0.13	0.29	−0.25	−0.09
TC2A	r	−0.35	0.47	−0.38	−0.48	0.30	−0.43	−0.15	0.35	−0.33	−0.07
TC3A	r	0.29	−0.49	0.33	0.39	0.16	0.35	0.47	−0.31	0.27	0.40
TCA	r	0.79 **	−0.70 *	0.75 **	0.79 **	0.02	0.71 **	0.61 *	−0.83 **	0.79 **	0.38
CEC	r	0.85 **	−0.74 **	0.81 **	0.84 **	−0.02	0.81 **	0.43	−0.81 **	0.85 **	0.10
CECA	r	0.96 **	−0.86 **	0.91 **	0.96 **	0.15	0.89 **	0.66 *	−0.92 **	0.96 **	0.33
STC1	r	−0.62 *	0.48	−0.55 *	−0.60 *	0.12	−0.56 *	−0.32	0.64 *	−0.60 *	−0.11
STC2	r	−0.72 **	0.64 **	−0.64 **	−0.71 **	0.06	−0.62 *	−0.46	0.73 **	−0.67 *	−0.27
STC3	r	0.56*	−0.46	0.54	0.55 *	−0.09	0.61 *	0.18	−0.44	0.56 *	−0.07
STC4	r	0.45	−0.31	0.38	0.42	0.27	0.36	0.19	−0.39	0.47	−0.01
STCA	r	0.79 **	−0.57 *	0.61 *	0.75 **	−0.07	0.64 *	0.35	−0.82 **	0.81 **	0.05

^1^ *, *p* < 0.05; **, *p* < 0.001. TC: Total carbohydrate peak area region and baseline, ca. 1189–909 cm^−1^; the three peaks at TC1, TC2, and TC3 were at the peak height of 1147, 1093, and 1029 cm^−1^, respectively. TC1A, TC2A, and TC3A: Peak area of peaks TC1, TC2, and TC3. TCA: Peak area of TC region. CEC: Cellulosic compound peak area region and baseline, ca. 1292–1189 cm^−1^; within this region, one major peak was centered at ca. 1240 cm^−1^. CECA: Peak area of CEC region. STC: Structural carbohydrate peak area region and baseline, ca. 1487–1189 cm^−1^; there were four peaks, STC1, STC2, STC3, and SCT4, which were centered at ca. 1461, 1423, 1371, and 1324 cm^−1^, respectively. STCA: Peak area of STC region. CB2, soluble fiber; CB3, digestible fiber; CC, indigestible fiber; CHO, total carbohydrate; NFC, non-fiber carbohydrate; aNDF, neutral detergent fiber; ADF, acid detergent fiber; ADL, acid detergent lignin.

**Table 5:** Correlation between the carbohydrate spectrum profiles and in situ degradation rates of and and ADF in rape straw ^1^.

Item		In Situ Rumen aNDF Degradation					In Situ Rumen ADF Degradation				
		*a*	*b*	*c*	*d*	*ED*	*a*	*b*	*c*	*d*	*ED*
TC1	r	0.49	0.54	−0.53	0.61 *	0.47	0.33	0.62 *	−0.45	0.60 *	0.43
TC2	r	0.70 *	0.65 *	−0.60 *	0.70 *	0.68 *	0.66 *	0.75 **	−0.56 *	0.78 **	0.72 **
TC3	r	0.32	0.06	−0.09	0.09	0.24	0.17	0.29	−0.06	0.35	0.26
TC1A	r	0.50	0.46	−0.44	0.53	0.48	0.35	0.59 *	−0.47	0.62 *	0.44
TC2A	r	0.43	0.46	−0.41	0.49	0.42	0.39	0.37	−0.25	0.35	0.43
TC3A	r	−0.14	−0.16	0.14	−0.18	−0.03	−0.25	0.20	0.08	0.24	−0.05
TCA	r	−0.56 *	−0.68 *	0.61 *	−0.69 *	−0.64 *	−0.70 *	−0.55 *	0.50	−0.53	−0.65 *
CEC	r	−0.58 *	−0.85 **	0.86 **	−0.85 **	−0.70 *	−0.81 **	−0.78 **	0.85 **	−0.77 **	−0.76 **
CECA	r	−0.67 *	−0.82 **	0.73 **	−0.81 **	−0.77 **	−0.97 **	−0.66 *	0.71 **	−0.67 *	−0.88 **
STC1	r	0.54	0.73 **	−0.63 *	0.75 **	0.84 **	0.66 *	0.91 **	−0.85 **	0.93 **	0.76 **
STC2	r	0.47	0.77 **	−0.63 *	0.75 **	0.82 **	0.73 **	0.86 **	−0.81 **	0.88 **	0.80 **
STC3	r	−0.46	−0.52	0.60 *	−0.55 *	−0.29	−0.42	−0.22	0.24	−0.13	−0.34
STC4	r	−0.20	−0.23	0.26	−0.24	−0.06	−0.30	0.07	−0.06	0.13	−0.15
STCA	r	−0.53	−0.81 **	0.83 **	−0.83 **	−0.59 *	−0.67 *	−0.70 *	0.71 **	−0.59 *	−0.59 *

^1^ *, *p* < 0.05; **, *p* < 0.001. *a*: The rapid degradable fraction in rumen degradation; *b*: the slowly degradable fraction in rumen degradation; *c*: the degradation rate of the slowly degradable fraction; *d*: the potentially degradable fraction in rumen degradation; *ED*: the effective degradability of the incubated samples. TC: Total carbohydrate peak area region and baseline, ca. 1189–909 cm^−1^; the three peaks, TC1, TC2, and TC3, were at the peak height of 1147, 1093, and 1029 cm^−1^, respectively. TC1A, TC2A, and TC3A: Peak area of peak TC1, TC2, and TC3. TCA: Peak area of TC region. CEC: Cellulosic compound peak area region and baseline, ca. 1292–1189 cm^−1^; within this region, one major peak was centered at ca. 1240 cm^−1^. CECA: Peak area of CEC region. STC: Structural carbohydrate peak area region and baseline, ca. 1487–1189 cm^−1^; there were four peaks, STC1, STC2, STC3, and SCT4, which were centered at ca. 1461, 1423, 1371, and 1324 cm^−1^, respectively. STCA: Peak area of STC region.

## Data Availability

Not applicable.

## Supplementary Materials

The following supporting information can be downloaded at https://www.mdpi.com/article/10.3390/ani13152421/s1. Table S1: Effect of different CaO dosages and moisture levels on spectral molecular structure of carbohydrates of rape straw.
