# Supplementary material for: Effect of Anaerobic Calcium Oxide Alkalization on the Carbohydrate Molecular Structures, Chemical Profiles, and Ruminal Degradability of Rape Straw
Source: Animals (Basel). 2023 Jul 26;13(15):2421. doi: 10.3390/ani13152421 (PMC10417835; doi:10.3390/ani13152421)
Supplement: Supplementary file 1 [file animals-13-02421-s001.zip › animals-2501968-supplementary.pdf]

Table S1. Effect of different CaO dosages and moisture levels on spectral molecular structure of carbohydrates of rape straw [1].

Item	Untreated Straw	3%		5%		7%		SEM[2]	*P*-value			Contrast[6]		
		50%M[7]	60%M	50% M	60% M	50% M	60% M		Moisture	CaO	M´CaO	Control vs Treated	L	Q
Total Carbohydrate (TC) Profiles[3]														
TC1	0.18	0.19	0.19	0.19	0.21	0.19	0.19	0.006	0.32	0.23	0.32	0.05	0.12	0.03
TC2	0.39^{a}	0.41^{ab}	0.41^{ab}	0.42^{ab}	0.43^{a}	0.42^{ab}	0.41^{ab}	0.006	0.49	0.12	0.31	0.01	0.01	0.05
TC3	0.72	0.71	0.72	0.72	0.74	0.73	0.71	0.014	0.76	0.61	0.56	0.89	0.89	0.93
TC1A	4.80	6.03	5.73	5.93	6.37	5.58	5.52	0.368	0.94	0.32	0.62	0.03	0.11	0.02
TC2A	16.8	18.3	17.4	16.9	19.2	17.9	18.2	0.795	0.43	0.94	0.20	0.23	0.24	0.45
TC3A	70.3	67.8	69.0	70.8	70.0	68.3	67.7	1.442	0.96	0.26	0.75	0.42	0.40	0.80
TCA	95.6^{a}	93.3^{ab}	93.3^{ab}	93.1^{ab}	92.1^{ab}	91.9^{ab}	91.4^{b}	1.227	0.39	0.13	0.82	0.05	<0.01	0.27
Cellulosic Compounds (CEC) Profiles[4]														
CEC	0.14^{a}	0.09^{b}	0.08^{b}	0.03^{c}	0.04^{c}	0.04^{c}	0.03^{c}	0.004	0.47	<0.01	0.18	<0.01	<0.01	<0.01
CECA	7.44^{a}	4.51^{b}	4.32^{b}	2.37^{c}	2.21^{c}	1.98^{c}	1.82^{c}	0.199	0.32	<0.01	1.00	<0.01	<0.01	<0.01
Structural Carbohydrate (STC) Profiles[5]														
STC1	0.08^{d}	0.11^{c}	0.11^{c}	0.16^{a}	0.15^{ab}	0.14^{abc}	0.13^{bc}	0.010	0.09	<0.01	0.46	<0.01	<0.01	<0.01
STC2	0.14^{c}	0.20^{ab}	0.18^{bc}	0.25^{a}	0.24^{a}	0.24^{ab}	0.24^{ab}	0.006	0.27	<0.01	0.68	<0.01	<0.01	<0.01
STC3	0.12^{ab}	0.11^{ab}	0.11^{ab}	0.12^{a}	0.11^{ab}	0.11^{ab}	0.10^{b}	0.004	0.02	0.10	0.36	0.07	0.03	0.82
STC4	0.10^{ab}	0.11^{ab}	0.11^{ab}	0.12^{a}	0.11^{ab}	0.10^{ab}	0.09^{b}	0.005	0.02	0.03	0.60	0.47	0.67	0.03
STCA	29.5^{a}	27.0^{ab}	26.1^{ab}	25.8^{ab}	25.1^{b}	26.0^{ab}	24.1^{b}	0.668	0.07	0.14	0.66	<0.01	<0.01	0.04

[1] Means with different letters within a row are significantly different at $P < 0.05$. [2]SEM, standard error of the mean. [3]Total carbohydrate (TC) peak area region and baseline, ca. 1189−909 cm^{-1} and the three peaks TC1, TC2, TC3 were at the peak height of 1147, 1093, and 1029 cm^{-1}, respectively; TC1A, TC2A, and TC3A: peak area of peak TC1, TC2, and TC3; TCA: peak area of TC region; [4]Cellulosic compounds (CEC) peak area region and baseline, ca. 1292−1189 cm^{-1}, and within this region one major peak was centered at ca. 1240cm^{-1}; CECA: peak area of CEC region; [5]Structural carbohydrate (STC) peak area region and baseline, ca. 1487−1189 cm^{-1}, there were four peaks STC1, STC2, STC3 and SCT4 which were centered at ca. 1461, 1423, 1371, and 1324 cm^{-1}, respectively; STCA: peak area of STC region. [6]L = linear; Q = quadratic. [7]M = moisture.
